# Coping, Anxiety, and Pain Intensity in Patients Requiring Thoracic Surgery

**DOI:** 10.3390/jpm11111221

**Published:** 2021-11-18

**Authors:** Elisei Moise Hasan, Crenguta Livia Calma, Anca Tudor, Cristian Oancea, Voicu Tudorache, Ioan Adrian Petrache, Emanuela Tudorache, Ion Papava

**Affiliations:** 1 Clinic of Thoracic Surgery, Emergency Clinical Municipal Hospital Timișoara, Gheorghe Dima Street No. 5, 300079 Timișoara, Romania; hasanelisei@yahoo.com (E.M.H.); ioan.petrache@umft.ro (I.A.P.); 2 University of Medicine and Pharmacy Timișoara, Eftimie Murgu Square No. 2, 300041 Timișoara, Romania; 3 Discipline of Physiology, Department of Functional Sciences, Center of Immuno-Physiology (CIFBIOTEH), “Victor Babeș” University of Medicine and Pharmacy Timișoara, Eftimie Murgu Square No. 2, 300041 Timișoara, Romania; 4 Discipline of Biostatistics and Medical Informatics, Department of Functional Sciences, “Victor Babeș” University of Medicine and Pharmacy Timișoara, Eftimie Murgu Square No. 2, 300041 Timișoara, Romania; atudor@umft.ro; 5 Discipline of Pneumology, Department of Infectious Diseases, “Victor Babeș” University of Medicine and Pharmacy Timișoara, Eftimie Murgu Square No. 2, 300041 Timișoara, Romania; oancea@umft.ro (C.O.); voicu.tudorache@yahoo.com (V.T.); emanuela.tudorache@umft.ro (E.T.); 6 First Discipline of Surgical Semiology, First Department of Surgery, “Victor Babeș” University of Medicine and Pharmacy Timișoara, Eftimie Murgu Square No. 2, 300041 Timișoara, Romania; 7 Discipline of Psychiatry, Department of Neuroscence, NEUROPSY-COG Center for Cognitive Research in Neuropsychiatric Pathology, “Victor Babeș” University of Medicine and Pharmacy Timișoara, Eftimie Murgu Square No. 2, 300041 Timișoara, Romania; papavaion@yahoo.com

**Keywords:** coping styles, distress, postoperative pain, thoracic surgery, quality of life

## Abstract

Stress, anxiety, and post-surgical chest pain are common problems among patients with thoracic surgical pathology. The way in which psychological distress is managed—the coping style—can influence the postsurgical evolution and quality of life of patients. In our study, we monitored the influence of coping style on patients’ anxiety and the intensity of post-operative chest pain. We conducted a cross-sectional study on 90 subjects with thoracic surgical pathology. One month after their surgeries, patients completed the following scales and questionnaires, translated, adapted, and validated for the Romanian population: COPE scale inventory, Generalized Anxiety Disorder-7 Questionnaire, McGill Pain Questionnaire, and Numeric Pain Rating Scale. Anxiety (evaluated using the Generalized Anxiety Disorder-7 Questionnaire) and postoperative thoracic pain intensity (evaluated by means of the Numeric Pain Rating Scale, Number of Words Chosen, and McGill Pain Questionnaire) were significantly higher in patients exhibiting social-focused coping than in patients presenting emotion-focused or problem-focused coping as their main coping style (Kruskal–Wallis, *p* = 0.028, *p* = 0.022, *p* = 0.042, *p* = 0.007). In our study, there were no differences observed in pain intensity relative to level of anxiety. Coping style is an important concept in the management of anxiety and pain experienced by patients undergoing chest surgery. Therefore, a multidisciplinary approach should be considered in clinical practice.

## 1. Introduction

Stress is a common interdisciplinary problem in medicine when patients are faced with serious medical problems, especially when discussing major surgeries [1]. Its negative effects can contribute to the emergence of certain pathologies or the unfavorable evolution of pre-existing conditions [2]. Thus, in the medical field, stress identification and how patients manage it has become necessary in improving the health status of hospitalized patients [3].

Among patients with an increased level of psychological distress are those with thoracic surgical pathology. Patients tend to overestimate their risk of lung cancer [4]. A patient requiring scheduled thoracic surgery has a fear of lung cancer of varying intensity, which may be relieved in whole or in part through the provision of information about the surgery, presumptive preoperative diagnosis (based upon computed tomography (CT)), and symptomatology [5]. Slatore et al. (2016) stated in their study of patients with incidental pulmonary nodules that some patients report psychological disturbance when faced with the news, with the most common forms being anxiety and depression. These responses are a defensive pathological reaction of the human body, and are hence termed pathological emotional responses [6]. Lack of attention to psychological anxiety and depression negatively impacts the physical and mental health of patients and even has repercussions on family members and society [7,8]. Some studies have suggested that the effects of anxiety and depression can lead to decreased quality of life and decreased chances of survival, by inhibiting immune function and lowering the pain threshold [9,10].

Preoperatively, psychological distress can lead to worsening pain, decreased postoperative cognitive function, and increased expenditure on medical services [11]. Anxiety and depression in the preoperative period can also compromise the surgical outcome. A high level of anxiety leads to increased excitability of the sympathetic nervous system, requiring higher doses of anesthetics and analgesics in the postoperative period. Depression may increase the incidence of postoperative infection and reduce patients’ stress capacity [12].

Furthermore, stress during surgery can generate anxiety; its presence has a negative impact on patients’ quality of life, as well as on their postoperative recovery. Many studies have indicated that more anxious patients have poorer outcomes, longer hospital stays, higher rate of complications, and prolonged convalescence [13]. Hence, much more attention should be focused on finding ways to reduce patient anxiety [14].

Postoperative chest pain also has a negative impact upon postoperative evolution, both in developing and maintaining anxiety [15]. In the case of thoracotomy, numerous studies have reported a prevalence between 33% and 91% of post-thoracotomy pain syndrome (PTPS) in patients who had undergone surgery. Arends et al. (2020) observed that the risk of developing PTPS is higher after open surgery (TT) than after video-assisted thoracic surgery (VATS) due to the extent of tissue and nerve damage. Moreover, analgesic treatment is often deficient and does not target neuropathic pain [16].

The exact mechanism underlying the development of chronic pain after thoracotomy is currently unknown, except for intraoperative nerve injuries. Otherwise, the age, sex, operative technique, existence of preoperative pain, genetic and psychosocial factors, and/or analgesic management are taken into consideration in the development of such chronic pain [17].

In fact, thoracotomy is considered one of the most painful medical procedures, and the pain resulting from it has been reported as acute, traumatic, and severe [18,19]. Therefore, postoperative pain in patients that have undergone thoracotomy is still a significant problem and still being investigated [20,21].

Psychological rehabilitation in thoracic surgical patients is still a concern for doctors, due to the psychological challenges generated by diagnosis, surgical treatment, and socio-professional reintegration. However, high-quality communication strategies have been demonstrated to improve patients’ knowledge of the risk of malignancy and to reduce emotional distress [22].

Coping is a psychological process that takes place consciously and is used to manage stressful life situations. Some coping styles may prove appropriate and adaptive in a stressful situation, while others may prove maladaptive to the same stressful situation and even have a seriously negative impact upon it. Recently, an increased interest in understanding the correlation between stress, symptom severity, and patient well-being has emerged in the literature [23]. While patients who use effective coping methods achieve a decrease in the symptoms of the disease and a better adaptation to it, those with poor coping may experience a worsening of symptoms, both physically and mentally [24,25,26].

Starting from the above-mentioned aspects, in our study we aimed to identify the main coping styles exhibited by patients with thoracic surgical pathology and their influence upon anxiety and on the intensity of chest pain felt by them in the postoperative period.

## 2. Materials and Methods

**Figure 1:**
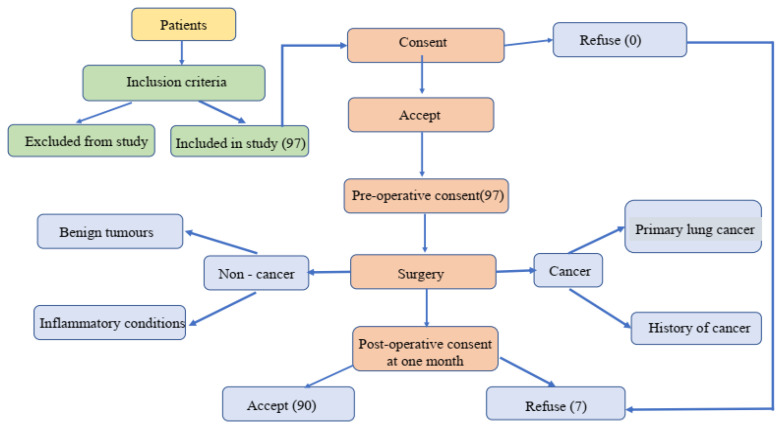
Flowchart of the patient enrollment process of study cohort.

In this cross-sectional, non-interventional study, we examined 90 patients with thoracic surgical pathology prior to and 1 month following their surgery. They were hospitalized and operated on in the Thoracic Surgery Department of the Municipal Emergency Hospital of Timisoara, Romania, between November 2018 and November 2019. Subjects were included in the study according to consecutive-case criteria and using a population-based approach. They were informed about the objectives and methods of the research and agreed to take part by providing written informed consent before and after surgery (Figure 1). The research protocol, investigation procedures, and informed consent form have the approval of the Ethics Committee of the Municipal Clinical Emergency Hospital of Timisoara and the Ethics Committee of “Victor Babes” University of Medicine and Pharmacy Timisoara.

Subjects were included in the study based on the following criteria:

- Patients over 18 years of age at the moment of inclusion in the research;
- Patients hospitalized at least 1 day before surgery;
- Patients diagnosed by imaging through chest CT that require a scheduled thoracic surgery;
- Patients who understood study procedures and agreed to take part and sign the Informed Consent Form (ICF).

The following were excluded from the study:

- Patients suffering from mental disorders that may interfere with the research methods and results;
- Patients unable to understand the research procedures or give written consent;
- Patients with stage IV lung neoplasm;
- Patients with multiple comorbidities and limited life expectancy;
- Thoracic surgical emergency.

Socio-demographic data of the patients were requested on the first day of hospitalization.

We offered all patients the opportunity to be included in our study according to the inclusion criteria presented. Postoperatively, we had 7 cases that refused, for various personal reasons, to complete the questionnaires; they were withdrawn from the study.

1. We used the COPE questionnaire to assess coping. Developed by Carver, Scheier, and Weintraub in 1989, the COPE scale is a self-report tool that evaluates the coping strategies of people to stress [27]. The questionnaire, validated and adapted for the Romanian population by Crașovan and Sava (2013), contains 60 items used in evaluating the 15 strategies and 4 coping styles [28]. The answer to each item can be ranked on a scale of 1 to 4, as follows: 1—usually do not do this, 2—rarely do this, 3—sometimes do this, 4—often do this.

The four coping styles analyzed with this tool were: coping focused on emotion, problem-focused coping, coping focused on social support, and avoidant coping. Each coping style included three coping mechanisms. For each patient, the coping style that presented the highest value compared to the other three was considered the dominant coping style.

2. The GAD-7 (General Anxiety Disorder-7) Questionnaire was employed to assess anxiety. The questionnaire includes seven items, with the answer from each item rated on a scale from 0 to 3. The final score is obtained by summing the scores of the seven items, with a higher score indicating greater anxiety severity. The sum of the seven items, and therefore the GAD-7 total score, will be a value between 0 and 21, with a total score of 5 representing mild anxiety, 10 representing moderate anxiety, and 15 representing severe anxiety [29].
3. The characteristics of pain were evaluated using the McGill Pain Questionnaire and Numeric Pain Rating Scale (NPRS).
  (a) The McGill Pain Questionnaire scale was utilized for the quantitative and descriptive assessment of the subjective sensation of pain. It consists of three major classes of words describing the painful experience (sensory, affective, and evaluative). It also contains a clinical pain intensity scale and other items to describe the painful sensation (miscellaneous). Pain intensity was rated on a scale from 0 to 5, whereby 0 represents the absence of pain and 5 is maximum intensity. The subscale Number of Words Chosen (NWC) was recorded following Melzack’s instructions [30].
  (b) The Numeric Pain Rating Scale (NPRS) is frequently applied in measuring the intensity of pain, with patients asked to select a number from 0 to 10 to indicate the severity of their pain [31].

Statistical data were presented using SPSSv17. In the analysis of numerical variables, average, minimum, and maximum values were estimated, as well as the variables’ standard deviations. The non-parametric Kruskal–Wallis test was applied for comparisons between more than two numerical series. For comparisons between two sets of values with no Gaussian distribution, the Mann–Whitney test was applied. The Student’s t-test was used in the case of two series of normally distributed values, while the chi-square (χ2) test was used for comparisons between nominal variables. The results were considered significant for a value of *p* < 0.05.

## 3. Results

Of the 90 patients, 24 were diagnosed with primary lung cancer and 16 had a history of cancer. Twenty-six patients presented benign tumor formations of variable size and were operated on by using a variety of minimally invasive or classic surgical procedures. Twenty-four patients were included postoperatively due to presenting inflammatory conditions. We analyzed the questionnaires, with the demographic characteristics of the 90 patients shown in Table 1. We observed that age was significantly older in neoplastic patients.

Depending on the type of surgical incision, patients were divided into three categories: thoracoscopic incision, mini-thoracotomy incision, and thoracotomy incision.

Table 2 shows that the type of surgery did not result in significant differences regarding the pain of patients evaluated at one month postoperatively or regarding their level of anxiety. The duration of thoracotomy surgery was significantly longer compared to the duration of thoracoscopy and mini-thoracotomy (Mann–Whitney test, *p* < 0.001).

Following the data obtained with the COPE scale, we analyzed the patients’ scores and grouped them into three categories, depending on the dominant coping type that they presented. Thus, patients were divided into those with coping focused on the problem (*n* = 37), coping focused on emotion (*n* = 33), and coping focused on social support (*n* = 20) data summarized in Table 3.

The coping scores showed a normal distribution for the entire study group (Shapiro–Wilk Test, *p* > 0.05) and are represented by their mean plus or minus one standard deviation: problem-oriented coping (35.87 ± 7.58), emotion coping (34.99 ± 5.94), and social-oriented coping (32.40 ± 6.78).

**Figure 2:**
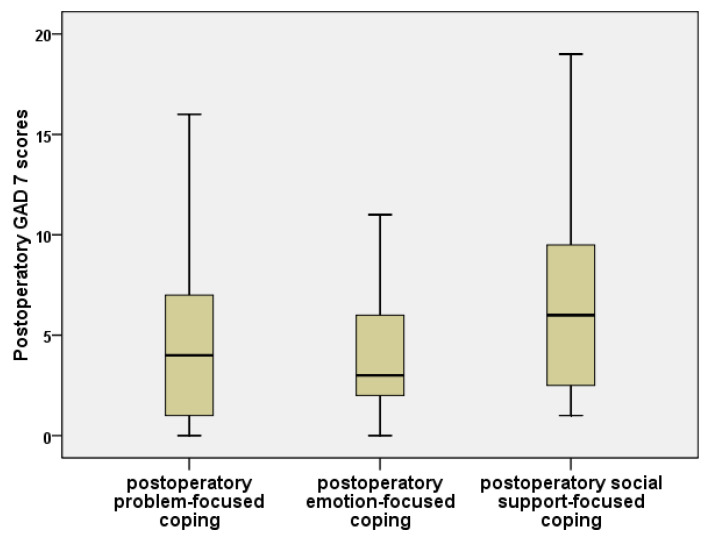
Boxplot for GAD-7 scores, compared by predominant postoperative type of coping.

Regarding anxiety, we noticed considerable differences between people from the three categories, namely, coping by focusing on the problem, on emotion, or on social support. GAD-7 anxiety values were significantly increased for social-support-oriented coping, in contrast to emotion- and problem-centered coping (Kruskal–Wallis, *p* = 0.028). Comparisons between the coping styles (Figure 2) also showed:

- The GAD-7 anxiety values were insignificantly higher for problem-focused coping than for emotional-focused coping (Mann–Whitney, *p* = 0.644);
- Problem-focused coping had significantly lower GAD-7 scores than coping centered upon social support (Mann–Whitney, *p* = 0.048);
- Emotion-focused coping presented significantly lower GAD-7 values than social-support coping (Mann–Whitney, *p* = 0.026).

**Figure 3:**
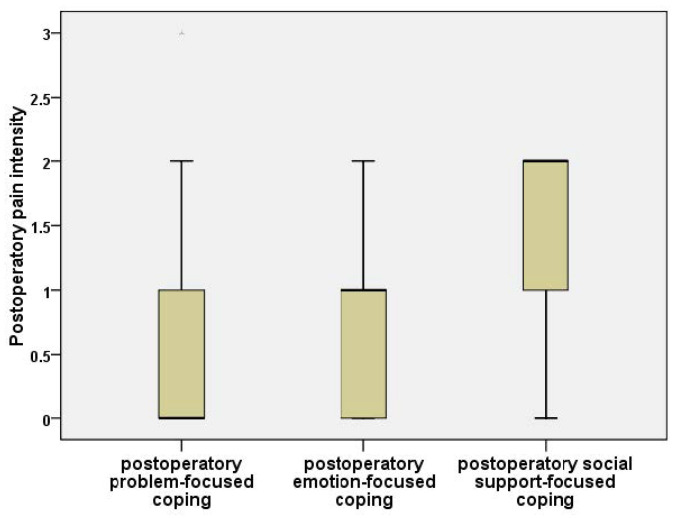
Boxplot for postoperative pain intensity measured with McGill Pain Questionnaire, compared by predominant postoperative type of coping.

Significant differences were established between the three subgroups for “total words” (Kruskal–Wallis, *p* = 0.042) and for pain intensity (Kruskal–Wallis, *p* = 0.007; McGill Pain Questionnaire). We refined the comparisons between the coping styles and obtained the following results (Figure 3):

- The differences between the scores obtained for the predominantly problem-centered coping style and predominantly emotion-centered coping style were insignificant, both in the case of “total words” (Mann–Whitney U Test, *p* = 0.458) and for the intensity of the pain (Mann–Whitney U Test, *p* = 0.619);
- The scores for “total words” were significantly increased in the predominantly social-support-centered coping cases in contrast to the problem-focused coping cases (Mann–Whitney U Test, *p* = 0.022). The intensity of pain was significantly higher in the social-support-oriented coping cases as opposed to the problem-oriented coping cases (Mann–Whitney U Test, *p* = 0.004);
- The scores for “total words” (Mann–Whitney U Test, *p* = 0.048) and the intensity of the pain (Mann–Whitney U Test, *p* = 0.006) were significantly higher in the predominant social-support-oriented cases compared with the emotion-focused coping cases.

**Figure 4:**
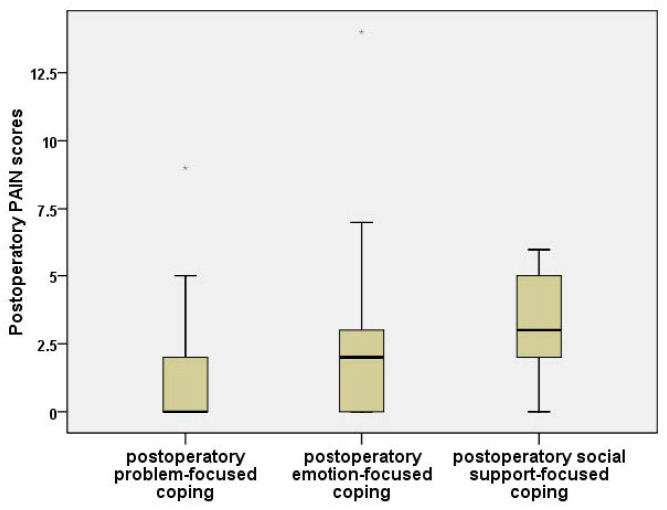
Boxplot for postoperative pain scores (NPRS), compared by predominant postoperative type of coping.

Postoperatively, the intensity of pain measured with NPRS was significantly increased for the social coping style compared to the other two styles (Kruskal–Wallis, *p* = 0.022). We compared the three coping styles (Figure 4) and observed that:

- The NPRS pain values were insignificantly lower for problem-directed coping in comparison to emotion-directed coping (Mann–Whitney, *p* = 0.362);
- The problem-focused coping NPRS scores were significantly lower than the social-support-directed coping scores (Mann–Whitney, *p* = 0.006);
- The emotion-focused coping values were significantly lower than the social-support-oriented coping values (Mann–Whitney, *p* = 0.042).

We divided the patients into four subgroups according to their GAD-7 values and obtained the following: 0–4 without anxiety (37 cases), 5–9 mild anxiety (33 cases), 10–14 moderate anxiety (8 cases), and 15–21 severe anxiety (6 cases) (Table 4).

There were no differences observed in pain intensity assessed one month postoperatively, relative to the intensity of anxiety.

## 4. Discussion

The results of our study indicate that any surgical approach (thoracotomy, mini-thoracotomy, or minimally invasive) generates postoperative pain of variable intensity depending upon the coping mechanism. Preoperative psychological evaluation of patients should be done regardless of the type of coping. Our study showed that patients with social-support-oriented coping had both a higher anxiety score and an increased intensity of post-surgical chest pain.

Furthermore, the intensity of pain felt by patients was not influenced by the intensity of anxiety, unlike most existing studies that conclude that a higher intensity of pain is dependent upon the presence or intensity of anxiety [32,33,34]. In the medical literature, evidence suggests that psychological factors play an important role in modulating pain experience, even for cancer patients [35].

One possible explanation is that in the above-mentioned studies, the selected groups were much more homogeneously outlined, such as groups of patients with coronary artery bypass graft or lung cancer. Conversely, our study presented a more diverse group of patients with thoracic-pulmonary pathology. Our research indicates that the way in which subjects handle stress—that is, the main coping style they use—influences both the intensity of anxiety and the intensity of pain they experience.

It is known that a patient’s distress may relate to their coping style when faced with challenging life events [36]. There are studies on surgical patients with chronic or oncological diseases, whereby the impact of coping style on the mental state, the potency of postoperative pain, the quality of life or recovery, as well as the postoperative evolution were analyzed, with heterogeneous results. We consider that some of the main causes for these heterogeneities include the distinctive definition of coping, the scales used, and the selection criteria for the groups. In general, the most common difference was the division of coping into active or passive coping and problem-focused or emotion-focused coping [37,38,39].

Our research used coping levels based upon an adaptation of the COPE scale on the Romanian population [27]. Therefore, those who presented a predominantly social-oriented coping obtained significantly higher values for the intensity of pain on the McGill scale and total words compared with the other two predominant coping styles. Amongst those exhibiting emotion-focused coping and those with problem-focused coping, there were no significant differences reported, both in terms of total words and pain intensity. Furthermore, upon evaluating the intensity of pain using the NPRS scale, it was found to be significantly more intense for patients with social-support-directed coping than for those with emotion-directed and problem-focused coping styles.

There have been situations described in the literature where the different styles of coping have influenced the pain in various analyzed aspects. Gil et al. (1989) observed a difference between patients with passive coping and active coping. The patients presenting a passive style of coping (catastrophizing) have asserted a higher intensity and frequency of pain than patients with active coping (self-verbalization and ignoring pain sensations). Hence, passive coping is maladaptive, while patients with active coping adapt better to painful situations and experience decreased pain intensity [40]. In Polanski’s study on patients with small cell lung carcinoma (SCLC), those who had a more active coping also had lower pain intensity [41]. Fisher et al. (2010) also observed a correlation between coping strategies and pain intensity [33]. A number of studies have purported that lung cancer patients most frequently present coping strategies focused on emotion, which are known to be less efficient than the strategies focused on the problem [23,42,43,44].

Moreover, Polanski et al. (2018) demonstrated that lung cancer patients with coping mechanisms that focused on the problem experienced less pain [45].

In our study, we observed that the intensity of pain did not differ significantly relative to the surgical approach performed. In addition, there were no significant differences between groups in terms of surgery time, taking the three surgical approach methods (thoracoscopy, mini-thoracotomy, and thoracotomy) into consideration. Hence, the influence of the dominant coping style is even more pertinent in understanding differences between patients in terms of anxiety and pain.

Regarding patients with non-small-cell lung carcinoma (NSCLC), studies indicate that focusing on emotions contributes to psychological distress. Consequences include both physical symptoms (e.g., nausea) and mental symptoms (e.g., depression) [46,47]. On the other hand, patients exhibiting strategies focused on the problem experience a decrease in the rate of depression, as well as in the intensity of other symptoms. However, they still struggle with anxiety [48].

In this research, the intensity of anxiety varied depending upon the dominant coping style. In our studied cases, the level of anxiety in patients with social-support-directed coping significantly surpassed that found in the other two coping categories (problem-centered coping and emotion-oriented coping).

There are extensive studies showing that coping has a significant role in handling anxiety [23,33,49]. In their research on patients with chronic obstructive pulmonary disease (COPD), Papava et al. noticed an association of coping style focused on social support with a higher level of anxiety [50,51]. Social-support-focused coping and avoidant coping, were associated with an increased level of anxiety in patients with lung cancer [49].

Cumulating our results with those mentioned in other studies, we suggest that for patients with somatic thoraco-pulmonary pathology, the coping style focused on social support has been most closely associated with high-intensity anxiety symptoms. 

There were no significant differences between those with emotion-centered coping and those with coping focused on the problem in terms of intensity of anxiety.

## 5. Conclusions

Coping may further impact the anxiety and pain experience associated with chest surgery pathology. According to our study, coping focused on social support is a less-adaptive coping mechanism than emotion-oriented or problem-focused coping types, both in terms of anxiety and pain.

The creation of a predictive preoperative algorithm could be an invaluable tool in quantifying the psychological impact upon the patient, while highlighting the need for tailored psychological care according to the subject’s coping mechanism. Future research shall demonstrate the need for the inclusion of psychotherapy in the “team board”.

Given their vulnerability, we recommend special care for patients exhibiting social-focused coping mechanism. Information about the surgical procedure and what to expect after the operation should be tailored according to the patient’s preferences regarding the type and amount of information. 

## Figures and Tables

**Table 1:** Demographic characteristics of the patients.

Variable	No Cancer (*n* = 50)	Cancer (*n* = 40)	*p*-Value
Age (mean ± std. dev.)	46.8 ± 13.29	62 ± 8.07	<0.001 *^,(a)^
Sex (Masculine) *n* (%)	24 (48%)	22 (55%)	0.532 ^(b)^
Marital Status (Yes) *n* (%)	39 (78%)	30 (75%)	0.805 ^(b)^
Higher education (Yes) *n* (%)	15 (30%)	6 (15%)	0.133 ^(b)^
With activity (student, employed) *n* (%)	30 (60%)	10 (25%)	0.001 *^,(b)^
Environment (urban) *n* (%)	30 (60%)	27 (67.5%)	0.514 ^(b)^
Smoke (Yes) *n* (%)	12 (24%)	10 (25%)	0.913 ^(b)^

*—significant difference; ^(a)^—unpaired t-test; ^(b)^—chi-square test.

**Table 2:** Subjects divided according to the type of surgical incision.

Variable	Thoracoscopy (*n* = 8)	Mini Thoracotomy (*n* = 27)	Thoracotomy (*n* = 55)	*p*-Value Kruskal–Wallis Test
GAD-7— postoperative	2 (4.8)	4 (6.0)	5 (6.0)	0.459
McGill—Sensory postoperative	0 (1.0)	1 (1.0)	1 (1.0)	0.392
McGill—Affective postoperative	0 (1.0)	1 (1.0)	1 (1.0)	0.370
McGill—Evaluative postoperative	0 (1.0)	1 (1.0)	1 (1.0)	0.392
McGill—Miscellaneous postoperative	0 (1.0)	1 (1.0)	1 (1.0)	0.392
McGill—“Total words” postoperative	0 (1.0)	1 (1.0)	1 (1.0)	0.430
McGill—Intensity of postoperative pain	0 (1.0)	1 (2.0)	1 (2.0)	0.377
McGill—Total postoperative	0 (1.0)	1 (1.0)	1 (1.0)	0.392
Numeric Pain Rating Scale (NPRS)	0 (2.0)	2 (4.0)	2 (3.0)	0.274
Duration of surgical intervention	90 (33.8)	90 (30.0)	180 (120.0)	<0.001 *

*—significant difference; the scores are represented by median (interquartile range) because of non-normal distribution of values (Shapiro–Wilk test, *p* < 0.05). Abbreviations: GAD-7, Generalized Anxiety Disorder-7; McGill, McGill Pain Questionnaire.

**Table 3:** Subjects divided according to their dominant coping style.

Variable	Coping Focused on Problem (*n* = 37)	Coping Focused on Emotion (*n* = 33)	Coping Focused on Social Support (*n* = 20)	*p*-Value Kruskal–Wallis Test
GAD-7	4 (7.0)	3 (5.0)	6 (8.0)	0.028 *
McGill—Sensory postoperative	0 (1.0)	1 (1.0)	1 (0.0)	0.134
McGill—Affective postoperative	0 (1.0)	1 (1.0)	1 (0.0)	0.152
McGill—Evaluative postoperative	0 (1.0)	1 (1.0)	1 (0.0)	0.134
McGill—Miscellaneous postoperative	0 (1.0)	1 (1.0)	1 (0.0)	0.134
McGill—“Total words” postoperative	0 (1.0)	1 (1.0)	1 (0.0)	0.042 *
McGill—Intensity of postoperative pain	0 (1.0)	1 (1.0)	2 (1.0)	0.007 *
McGill—Total postoperative	0 (1.0)	1 (1.0)	1 (0.0)	0.134
Numeric Pain Rating Scale (NPRS)	0 (3.0)	2 (3.0)	3 (3.0)	0.022 *
Duration of surgical intervention	120 (130.0)	115 (118.0)	117.5 (83.0)	0.993

*—significant difference; the scores are represented by median (interquartile range) because of non-normal distribution of values (Shapiro–Wilk test, *p* < 0.05). Abbreviations: GAD-7, Generalized Anxiety Disorder-7; McGill, McGill Pain Questionnaire.

**Table 4:** Subjects divided according to GAD-7 anxiety values.

Variable	Without Anxiety (*n* = 37)	Mild Anxiety(*n* = 33)	Moderate Anxiety (*n* = 8)	Severe Anxiety (*n* = 6)	*p*-Value Kruskal–Wallis Test
NPRS postoperative	2 (3.0)	1 (3.0)	0 (4.0)	3.5 (5.0)	0.253
McGill—Sensory postoperative	1 (1.0)	0.5 (1.0)	0 (1.0)	1 (1.0)	0.186
McGill—Affective postop	1 (1.0)	0.5 (1.0)	0 (1.0)	1 (2.0)	0.178
McGill—Evaluative postoperative	1 (1.0)	0.5 (1.0)	0 (1.0)	1 (1.0)	0.186
McGill—Intensity of postoperative pain	1 (2.0)	0.5 (2.0)	0 (1.0)	1 (2.0)	0.160
McGill—Total postoperative	1 (1.0)	0.5 (1.0)	0 (1.0)	1 (1.0)	0.186

The scores are represented by median (interquartile range) because of non-normal distribution of values (Shapiro–Wilk test, *p* < 0.05).

## Data Availability

The data presented in this study are available upon request from the corresponding author. The data are not publicly available because the database contains patient personal data.
